# Circulating levels of Meteorin-like protein in polycystic ovary syndrome: A case-control study

**DOI:** 10.1371/journal.pone.0231943

**Published:** 2020-04-24

**Authors:** Fatima Zahraa Fouani, Reza Fadaei, Nariman Moradi, Zahra Zandieh, Soheila Ansaripour, Mir Saeed Yekaninejad, Akram Vatannejad, Maryam Mahmoudi

**Affiliations:** 1 Department of Cellular and Molecular Nutrition, School of Nutritional Sciences and Dietetics, Tehran University of Medical Sciences, Tehran, Iran; 2 Sleep Disorders Research Center, Kermanshah University of Medical Sciences, Kermanshah, Iran; 3 Cellular and Molecular Research Center, Research Institute for Health Development, Kurdistan University of Medical Sciences, Sanandaj, Iran; 4 Endocrine Research Center, Institute of Endocrinology and Metabolism, Iran University of Medical Sciences, Tehran, Iran; 5 Cellular and Molecular Research Center, School of Medicine, Iran University of Medical Sciences, Tehran, Iran; 6 Shahid Akbar Abadi Clinical Research Development Unit (ShACRDU), Iran University of Medical Sciences, Tehran, Iran; 7 Reproductive Biotechnology Research Center, Avicenna Research Institute, ACECR, Tehran, Iran; 8 Department of Statistics, School of Public Health, Tehran University of Medical Sciences, Tehran, Iran; 9 Department of Comparative Biosciences, Faculty of Veterinary Medicine, University of Tehran, Tehran, Iran; 10 Student’s Scientific Research Center, Tehran University of Medical Sciences, Tehran, Iran; Zhejiang University School of Medicine Women's Hospital, CHINA

## Abstract

Patients diagnosed with polycystic ovary syndrome (PCOS) are at high risk of developing a myriad of endocrinologic and metabolic derailments. Moreover, PCOS is a leading cause of habitual abortion, also known as recurrent pregnancy loss (RPL). Meteorin-like protein (Metrnl) is a newly discovered adipokine with the potential to counteract the metaflammation. This study aimed at determining the associations of serum Metrnl levels with homocysteine, hs-CRP, and some components of metabolic syndrome in PCOS-RPL and infertile PCOS patients.This case-control study was conducted in 120 PCOS patients (60 PCOS-RPL and 60 infertile) and 60 control. Serum hs-CRP and homocysteine were assessed using commercial kits, while adiponectin, Metrnl, FSH, LH, free testosterone and insulin levels were analyzed using ELISA technique. Serum Metrnl levels were found to be lower in PCOS patients when compared to controls (67.98 ± 26.66 vs. 96.47 ± 28.72 pg/mL, P <0.001*)*). Furthermore, serum adiponectin levels were lower, while free testosterone, fasting insulin, HOMA-IR, homocysteine, and hs-CRP were significantly higher in PCOS group compared to controls. Moreover, serum Metrnl correlated with BMI, adiponectin, and homocysteine in controls, and inversely correlated with FBG, fasting insulin, and HOMA-IR in PCOS group and subgroups. Besides, it inversely correlated with hs-CRP in control, and PCOS group and subgroups. These findings revealed a possible role of Metrnl in the pathogenesis of PCOS and RPL. Nevertheless, there is a necessity for future studies to prove this concept.

## Introduction

Infertility, or the inability to conceive for more than twelve months, is a highly prevalent reproductive problem, affecting 8–12% of reproductive-aged couples globally [1]. More than 50% of the cases are related to male gender irregularities; however, infertility continues to be a female’s social burden [2].

Polycystic ovary syndrome (PCOS) is one of the most prevalent ovulatory disorders contributing to infertility. It is a common polygenic, multifactorial, inflammatory, endocrine disorder, which evinces mainly due to lifestyle factors such as low-fiber high-fat diet, sedentary lifestyle, smoking and alcohol consumption [3–7], with a global prevalence of about 20% of women of reproductive age [8]. It is characterized by disrupted neuroendocrine mechanisms and a vast degree of symptomatic heterogeneity in ovulatory dysfunction, functional hyperandrogenism, and polycystic ovary morphology (PCOM) [9]. Females affected by PCOS may suffer from fertility challenges, oblivious to their ovulatory disorder until they become diagnosed with habitual abortion, also known as recurrent pregnancy loss (RPL)–defined as at least two consecutive abortions within the first 20 weeks of pregnancy, with an incidence of 1 in 300 pregnancies [10]. The pathogenesis of PCOS shares several features with metabolic syndrome (MetS), including dysfunctional adipose tissue with visceral adiposity, impaired insulin action with an increased risk for developing type II diabetes mellitus (T2DM), pro-atherogenic dyslipidemia, non-alcoholic fatty liver disease (NAFLD), and metaflammation [11–14].

Adipose tissue is not a mere fat depot; it is a metabolically active organ, releasing several cytokines, known as adipokines, regulating metabolic homeostasis and immune response. As a result of excess energy balance, the tissue cell mass expands. Metabolic dysregulation occurs whenever the expansion rate exceeds vascularization status. A sequela of impaired angiogenesis, hypoxia, cell death, and fibrosis take place, attracting macrophages and other immune cells, with an upregulation of pro-inflammatory (tumor necrosis factor (TNF)-α, Interleukin (IL)-6) and pro-fibrogenic factors (collagen and lysyl oxidase (LOX)). This eventually leads to a state of metaflammation [15, 16], and dysregulated release of adipokines, coercing mesenchymal stem cells to commit to the adipocyte lineage [17]. This pathology is the link between excess adiposity and number of disorders, including osteosarcopenic obesity [18], insulin resistance, T2DM [19], tumors [20], etc. Ongoing research revealed that adipocyte dysfunction is pivotal in the pathogenesis of ovulatory disorders in females [21], including PCOS [22].

Meteorin-like protein (Metrnl) is a novel immunoregulatory adipokine, widely expressed by white adipocytes, activated monocytes and macrophages [23, 24]. It promotes the differentiation of functional adipocytes and the browning of white adipocytes upon thermogenic stimulus, antagonizes insulin resistance, and suppresses inflammatory immune response [25].

Few studies have investigated Metrnl in the clinical setting [24, 26–28]. Much of the information is derived from animal studies, and none of them considered a possible relation between Metrnl and MetS within the pathogenesis of PCOS. The focus of this study is to investigate a possible association between Metrnl with components of MetS and cardiovascular biomarkers, and its relation with the pathogenesis of PCOS and RPL.

## Materials and methods

### Study design, setting, and participants

The study was conducted under the Declaration of Helsinki and was approved by the Ethical Committee of Tehran University of Medical Sciences, Tehran, Iran. All enrolled subjects signed a written consent form. Subjects were selected from the Obstetrics and Gynecology Department of Ibn Sina Infertility Center, Tehran, Iran, and controls were recruited from those performing routine checkups in the laboratory at the same center, from May 2017 to Jan 2018. The subjects were previously involved in our studies on PCOS and adipokines, and some of their data have been utilized in a recent publication [29].

This case-control study included 60 PCOS-RPL, 60 infertile PCOS (PCOS-Inf) and 60 control aged between 20–40 years, as described previously (published data) [29]. The inclusion criterion included PCOS diagnosis according to the 2003 Rotterdam Criteria [30], which dictates that two out of the following three characteristics should be met, PCOM on ultrasound, clinical and/or biochemical hyperandrogenism, and oligo- or ano-vulation (oligo-amenorrhea), with differential diagnosis of conditions such as hyperprolactinemia, thyroid diseases, premature ovarian failure, congenital adrenal hyperplasia, Cushing’s syndrome, and adrenal tumors. PCOM was defined as presence of at least 12 follicles with a diameter of 2–9 mm in each ovary and/or increased ovarian volume (at least 10 mm ^3)^, detected by sonographic imaging. Hyperandrogenism was defined as clinical symptoms (hirsutism with modified Ferriman-Gallwey score exceeding 8) and/or biochemical symptoms with an increment in serum free testosterone levels (exceeding 0.6 pg/mL) [30, 31]. Chronic oligo- and/or amenorrhea (oligo- or anovulation) were defined as infrequent menstruation with menstrual cycle exceeding 35 days, with less than eight cycles per year. Subjects designated for the PCOS-RPL group were those who simultaneously had at least two consecutive miscarriages before their 20^th^ week of gestation [32]. Infertile patients were those who were unable to conceive after one year of unprotected intercourse as a consequence of PCOS pathogenesis. Complete evaluation of all PCOS subjects were performed. Females with other causes of infertility such as tubal factor infertility, endometriosis, and anatomical abnormalities in the reproductive tract were excluded. The control group included fertile subjects with regular menstrual cycles and had no clinical/biochemical hyperandrogenism. Subjects who smoke, are pregnant, are lactating, have gynecological or obstetric problems, are receiving hormonal therapy, have viral, bacterial, or inflammatory disease, or suffer from CVD, thyroid diseases, or DM were excluded. Moreover, subjects who have been taking the following pharmaceutical products for the past six months were excluded: glucocorticoids, prescription weight loss drugs, estrogenic and anti-androgenic drugs, and anti-hypertensive drugs.

### Variable measurements

#### Anthropometrics and biochemical measurements

Anthropometric data, lifestyle factors, and medications were obtained for each subject. BMI was calculated using a standard formula [weight (kg)/ height (m^2^)]. Five milliliters of venous blood were collected from each subject after overnight fasting at the follicular phase of their menstrual cycle. FBG, lipid profile (serum triglyceride (TG), total cholesterol (TC), LDL-C, HDL-C), homocysteine, hs-CRP, fasting insulin, free testosterone, FSH, and LH were measured as previously described [29]. The homeostatic model assessment of insulin resistance (HOMA-IR) was calculated using the following equation: [(FBG (mg/dL)] × [fasting blood insulin (μU/mL)] / 405 [33].

#### Measuring adipokines

Serum levels of adiponectin were measured using ELISA technique, as previously described [29]. Commercial ELISA kits were utilized to measure serum levels of Metrnl protein (Aviscera Biosciences, U.S.A.). Moreover, the intra- and inter-assay coefficients of variation (CV) of Metrnl were 5% and 4.67%, respectively.

### Bias

Selection bias was addressed by closely matching cases to controls based on age and BMI.

### Statistical methods and sample size calculation

Statistical analysis was performed using IBM SPSS Statistics 20.0 (IBM SPSS, Chicago, IL, U.S.A.). Categorical variables were presented in frequency and percentage, and compared by Chi-square test. The normality of the continuous variables was checked using Kolmogorov-Smirnov test. Normal variables were presented by mean and standard deviation (SD) and compared using Student’s T test or one-way ANOVA with Bonferroni test. Skewed data were represented as median and quartile ranges, and compared using Mann-Whitney U test or Kruskal-Wallis tests, with Bonferroni corrections post hoc test. The effect of confounders (age, BMI, adiponectin levels, and metformin administration) on serum Metrnl was adjusted using Analysis of covariance (ANCOVA). Before heading toward further analysis, the logarithms of nonparametric data were calculated to approximate normality. The correlation between serum Metrnl with anthropometric and metabolic variables was tested using Pearson correlation test. Multiple linear regression was performed with all correlated parameters with serum Metrnl. The association of serum Metrnl with the risk of PCOS and RPL was assessed by multinomial logistic regression. All tests were two-sided, and a p-value of less than 0.05 was considered of statistical significance.

The sample size was determined on the basis of obtaining a significant difference of serum Metrnl level between two groups of 15 pg/mL, with an estimated SD of 23 pg/mL in each group, 95% statistical power, and 5% type I error [34].

## Results

### Characteristics of the study population

Table 1 lists the characteristics of the study population. When comparing controls to the PCOS group, there was no significant difference in terms of age, BMI, FBG, TG, TC, LDL-C, HDL-C, LH, FSH, and LH to FSH ratio levels. However, the PCOS group, compared to controls, had significantly higher levels of free testosterone, fasting insulin, and HOMA-IR.

**Table 1:** Clinical features of the study pulation.

Variables	Control group (n = 60)	PCOS group (n = 120)	PCOS-Inf subgroup (n = 60)	PCOS-RPL subgroup (n = 60)	p-value
**Age (years)**	30.02 ± 4.60	29.88 ± 4.22	29.88 ± 4.23	29.88 ± 4.23	0.981
**BMI (Kg/m**^**2**^**)**	25.48 ± 3.26	26.01 ± 3.39	25.86 ± 3.53	26.17 ± 3.25	0.536
**FBG** (mg/dL)	91.15 ± 9.68	90.62 ± 10.76	90.92 ± 11.6	90.32 ± 9.95	0.903
**TG** (mg/dL)	122.5 [97.5–155.0]	126.5 [92.5–165.5]	111.5 [82.5–142.0]	145.0 [103.25–175.25] ^d^**	0.009
**TC** (mg/dL)	163.84 ± 39.09	173.61 ± 35.58	171.82 ± 33.70	175.40 ± 37.57	0.216
**LDL-C** (mg/dL)	96.80 ± 30.21	100.84 ± 29.26	102.48 ± 26.81	99.20 ± 31.66	0.575
**HDL-C** (mg/dL)	46.09 ± 6.91	45.00 ± 9.93	44.93 ± 8.69	45.06 ± 11.11	0.744
**FSH** (IU/L)	6.43 ± 2.40	7.00 ± 3.30	7.00 ± 4.02	7.00 ± 2.40	0.500
**LH** (IU/L)	8.56 ± 2.38	9.38 ± 5.01	10.08 ± 3.95	8.68 ± 5.84	0.099
**Free Testosterone** (pg/mL)	1.50 ± 0.34	3.19 ± 1.10 ^a^**	3.15 ± 0.90 ^b^**	3.24 ± 1.28 ^c^**	< 0.001
**Log LH to FSH ratio**	1.29 [0.96–1.92]	1.26 [0.92–1.90]	0.40 [0.10–0.70]	0.08 [-0.34–0.54] ^d^**	0.009
**Fasting Insulin** (μU/mL)	2.94 [2.12–4.16]	3.71 [2.61–6.43] ^a^*	3.77 [2.93–5.37] ^b^*	3.32 [2.20–7.34] ^c^*	0.008
**HOMA-IR**	0.62 [0.45–0.97]	0.78 [0.61–1.33] *	0.80 [0.61–1.24] ^b^*	0.74 [0.53–1.76] ^c^*	0.012
**Metformin** (%)	1 (1.5)	66 (65.3)	28 (41.8)	38 (56.7)	< 0.001

Categorical data are given in frequency and percentage

Parametric data are given as mean ± standard deviation

Non-parametric data are given as median and interquartile range [25–75%]

Post Hoc Analysis are given as mean and median of each group on the far-right column

^a^ Comparison between Control group and PCOS group

^b^ Comparison between Control group vs PCOS-inf subgroup

^c^ Comparison between Control group and PCOS-RPL subgroup

^d^ Comparison between PCOS-inf and PCOS-RPL subgroups

* P < 0.05 is of statistical significance

** P < 0.001 is of statistical significance

PCOS: Polycystic ovary syndrome; RPL: Recurrent pregnancy loss; PCOS-inf: infertile PCOS; BMI: Body mass index; FBG: fasting blood glucose; TG: triglyceride; TC: total cholesterol; LDL-C: Low-density lipoprotein cholesterol; HDL-C: High-density lipoprotein cholesterol; LH: luteinizing hormone; FSH: follicle-stimulating hormone; HOMA-IR: Homoeostasis Model Assessment of Insulin Resistance

Furthermore, PCOS-Inf, PCOS-RPL, and control subjects were similar in terms of age, BMI, TC, LDL-C, and HDL-C. However, serum TG was significantly higher in PCOS-RPL than in infertile PCOS patients. Concerning the hormonal profile, PCOS-Inf subgroup had higher LH to FSH ratio and free testosterone levels, when compared to PCOS-RPL subgroup. Despite similar serum levels of FBG, PCOS-Inf and PCOS-RPL subgroups had significantly higher levels of fasting insulin and HOMA-IR, when compared to the control group, with higher levels in the infertile subgroup. These findings were similar to those in our previous publication [29].

The whole sample was stratified according to BMI stages: normal weight (BMI < 25 Kg/m^2^) and overweight/obese (BMI ≥ 25 Kg/m^2^) for further analysis. Overweight/obese subjects exhibited significantly lower levels of serum HDL-C in the PCOS-RPL subgroup, lower levels of FSH in the PCOS group and infertile subgroup, and higher levels of fasting insulin in the PCOS group and PCOS-inf subgroup, when compared to their normal-weight counterparts (P < 0.05 for all). Moreover, they had significantly lower levels of serum adiponectin in all groups (P < 0.05), except for the PCOS-RPL subgroup, although not statistically significant. (S1 Table)

### Serum levels of cardiovascular biomarkers and adipokines

**Fig 1:**
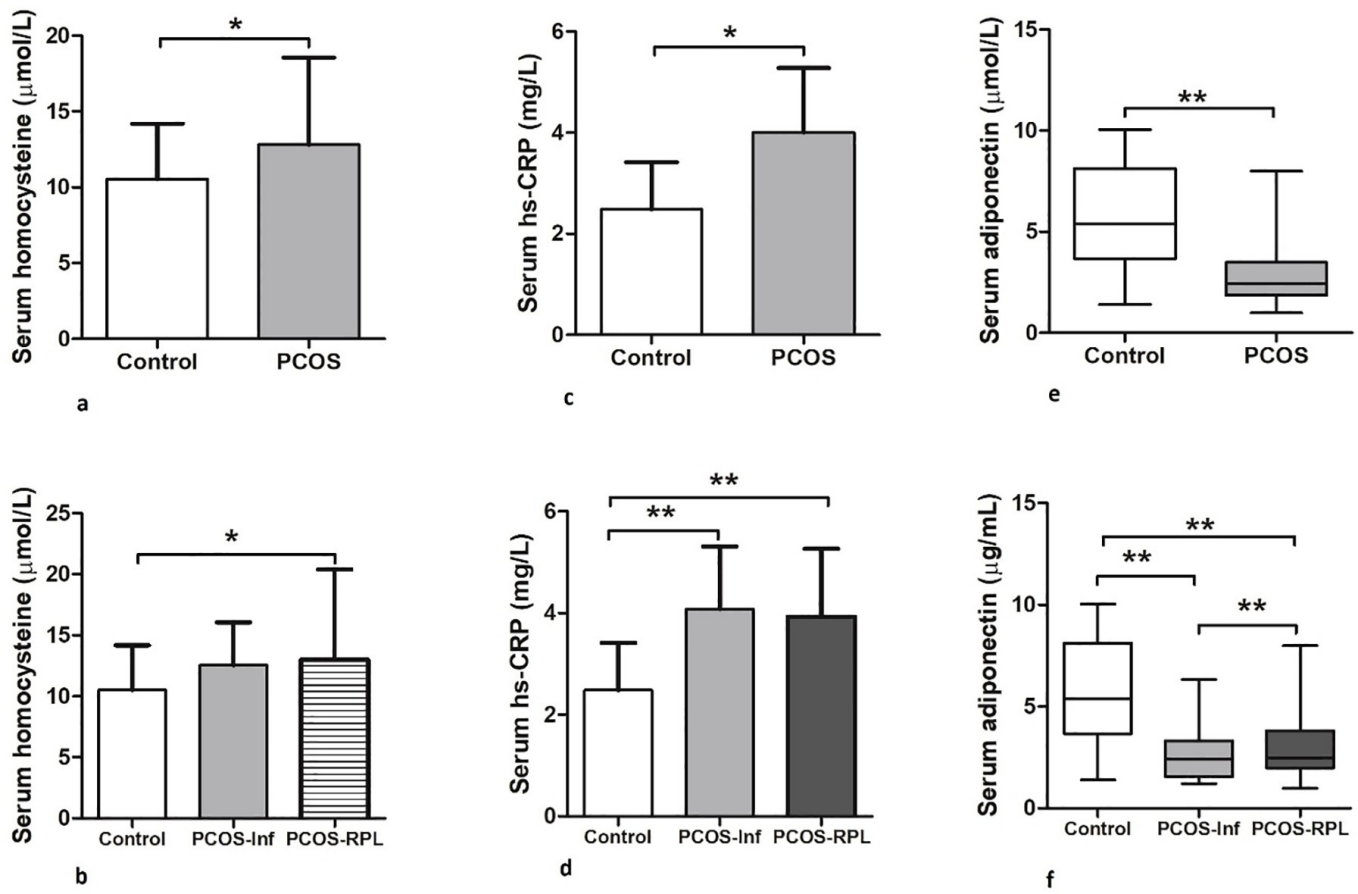
Serum levels of cardiovascular biomarkers and adiponectin. (a) Comparison of serum levels of homocysteine (μmol/L) in control vs. PCOS group (P < 0.05), and (b) control vs. PCOS-Inf vs. PCOS-RPL subgroups (P < 0.05). (c) Comparison of serum levels of hs-CRP (mg/L) in control vs. PCOS group (P < 0.05), and (d) control vs. PCOS-Inf vs. PCOS-RPL subgroups (P < 0.001). (e) Comparison of serum levels of adiponectin (μmol/L) in control vs. PCOS group (P < 0.001), and (f) control vs. PCOS-Inf vs. PCOS-RPL subgroups (P < 0.001). *PCOS*: *Polycystic ovary syndrome; PCOS-Inf*: *Infertile PCOS; RPL*: *Recurrent pregnancy loss; hs-CRP*: *high sensitivity C-reactive protein*.

The serum levels of homocysteine were significantly higher in the PCOS group and PCOS-RPL subgroup when compared to control. Furthermore, serum hs-CRP levels were significantly higher in the PCOS group and subgroups, when compared to the control group. (Fig 1)

**Fig 2:**
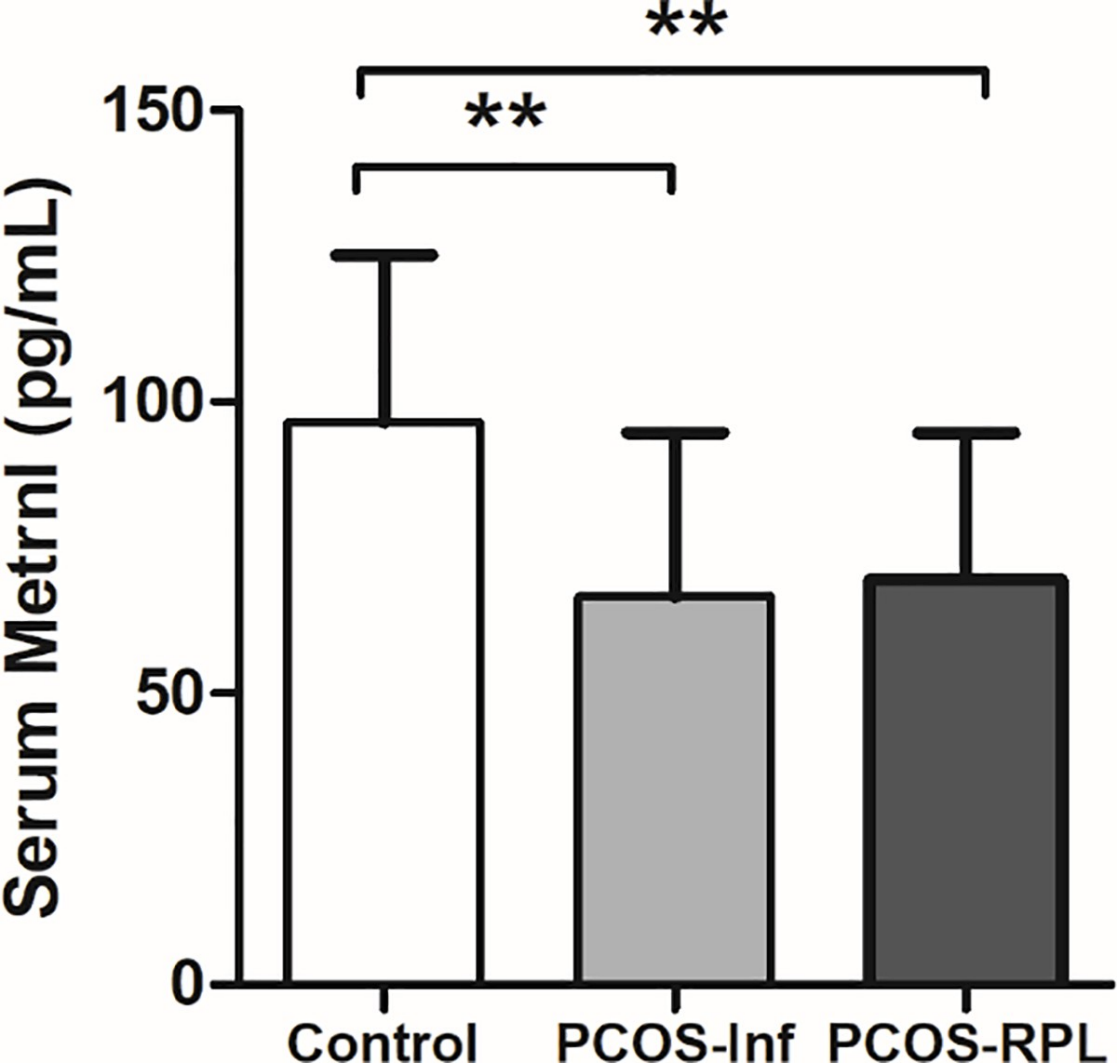
Serum levels of Metrnl protein (pg/mL) in control vs. infertile PCOS vs. PCOS-RPL subgroups (96.47 ± 28.72, 66.57 ± 28.14, and 69.39 ± 25.25, respectively, P < 0.001). PCOS: Polycystic ovary syndrome; PCOS-Inf: Infertile PCOS; RPL: Recurrent pregnancy loss.

On the other hand, serum adiponectin levels were significantly lower in the PCOS group and subgroups, when compared to controls. These results were similar to those we have published previously [29]. Likewise, serum levels of Metrnl were significantly lower in PCOS group (67.98 ± 26.66 pg/mL), PCOS-Inf (66.57 ± 28.14 pg/mL) and PCOS-RPL subgroups (69.39 ± 25.25 pg/mL), when compared to controls (96.47 ± 28.72 pg/mL) (P < 0.001 for all) (Fig 2). Following adjustment for the covariates (age, BMI, serum adiponectin, and metformin), the difference remained significant in serum Metrnl levels between control and PCOS groups (P < 0.001). It is noteworthy to point that no significant difference was observed in the levels of the three variables among the PCOS subgroups.

The difference in serum Metrnl protein between the groups was further tested after stratification by BMI, into normal weight (BMI < 25 Kg/m^2^) against overweight/obese (BMI ≥ 25 Kg/m^2^) (Table 2). Serum Metrnl levels showed no significant difference between overweight/obese individuals than normal-weight in the control group, PCOS-Inf and PCOS-RPL groups. Intriguingly, overweight/obese subjects demonstrated lower Metrnl levels compared to normal weight subjects in whole sample and PCOS groups.

**Table 2:** Mean comparison of serum Metrnl levels in normal (< 25kg/m^2^) and overweight/obese (≥ 25 kg/m^2^) patients.

Groups	BMI Staging	Number of Cases	Metrnl (pg/mL)	p-value
**Whole Sample** (n = 180)	Normal Weight	79	82.19 ± 31.89	0.008
	Overweight/Obese	101	742.21 ± 28.28	
**Control group** (n = 60)	Normal Weight	26	103.87 ± 27.78	0.080
	Overweight/Obese	34	90.80 ± 28.52	
**PCOS group** (n = 120)	Normal Weight	53	74.54 ± 29.43	0.019
	Overweight/Obese	67	62.79 ± 23.17	
**PCOS-Inf subgroup** (n = 60)	Normal Weight	27	72.35± 30.45	0.152
	Overweight/Obese	33	61.84 ± 25.59	
**PCOS-RPL subgroup** (n = 60)	Normal Weight	26	76.82 ± 28.75	0.056
	Overweight/Obese	34	63.70 ± 20.90	

The data are represented as mean ± standard deviation (SD).

PCOS: Polycystic ovary syndrome; PCOS-Inf: Infertile PCOS; RPL: Recurrent pregnancy loss; BMI: Body mass index

### Association of serum Metrnl with clinical parameters

Correlations between serum Metrnl level and other clinical parameters were analyzed (Table 3). Serum Metrnl levels inversely correlated with BMI and circulating LH levels in the control group and PCOS-RPL group. In the PCOS group and subgroups, it negatively associated with the parameters of glucose homeostasis: FBG, log fasting insulin, and log HOMA-IR. No associations were detected between serum Metrnl levels and age, serum levels of TC, LDL-C, HDL-C, LH, and FSH, and LH to FSH ratio in PCOS subgroups.

**Table 3:** Correlation of serum Metrnl protein with anthropometric, hormonal and biochemical variables.

Variables	Control group (n = 60)	PCOS group (n = 120)	PCOS-Inf subgroup (n = 60)	PCOS-RPL subgroup (n = 60)
**BMI** (Kg/m^2^)	- 0.347**	- 0.138	- 0.011	- 0.297*
**FBG** (mg/dL)	- 0.230	- 0.314**	- 0.318*	- 0.306*
**Log TG**	0.017	- 0.118	- 0.270*	0.015
**LH** (IU/L)	- 0.277*	0.086	0.072	0.117
**Log Fasting Insulin**	- 0.174	- 0.248**	- 0.278*	- 0.236
**Log HOMA-IR**	- 0.199	- 0.293**	- 0.319*	- 0.281*
**hs-CRP** (mg/L)	-0.283*	-0.365**	-0.364**	- 0.365**
**Homocysteine** (μmol/L)	- 0.214	- 0.028	- 0.012	- 0.043
**Log Adiponectin**	0.323*	- 0.012	- 0.124	0.086

Pearson correlation analyses were performed to determine if an association exists between the variables.

* P < 0.05

** P < 0.01

PCOS: Polycystic ovary syndrome; PCOS-Inf: Infertile PCOS; RPL: Recurrent pregnancy loss; BMI: Body mass index; FBG: fasting blood glucose; TG: triglyceride; LH: luteinizing hormone; HOMA-IR: Homoeostasis Model Assessment of Insulin Resistance; hs-CRP: high sensitivity C-reactive protein

Furthermore, a positive correlation was noted with log serum adiponectin in the control group only. On the other hand, an inverse correlation existed between serum Metrnl and hs-CRP levels in all groups, but with a higher statistical significance in the PCOS group and subgroups, as compared to controls. However, an inverse correlation between serum Metrnl and homocysteine was observed only in the whole sample (r = - 0.153, P = 0.040), which remained valid even after adjustment for folic acid intake (P = 0.039).

Next, multiple linear regression models were used to validate the prediction of serum Metrnl protein with those variables that had previously showed significant correlations. A multiple regression was operated to predict serum Metrnl levels from BMI, FSH, hs-CRP and log serum adiponectin in the control group. BMI (- 0.268, 95% CI [- 4.52—- 0.20]), serum hs-CRP (-0.260, 95% CI [- 15.24—- 0.72]) and LH (- 0.347, 95% CI [- 6.98—- 1.40]) significantly predicted serum Metrnl (P < 0.05). In PCOS group, FBG (1.965, 95% CI [0.70–9.04]), log fasting insulin (10.650, 95% CI [96.89–855.06]), log HOMA-IR (- 11.598, 95% CI [- 866.33—- 106.15]) and serum hs-CRP (- 0.321, 95% CI [- 10.09—- 3.24]) significantly predicted serum Metrnl (P < 0.05). Serum hs-CRP (- 0.328, 95% CI [- 13.25—- 1.71]) and log serum adiponectin (- 0.328, 95% CI [- 38.05—- 5.49]) significantly predicted serum Metrnl in the PCOS-Inf subgroup (P < 0.05). Finally, in the PCOS-RPL, serum hs-CRP (- 0.291, 95% CI [- 10.09—- 0.89]) and log serum HOMA-IR (- 0.243, 95% CI [- 17.36—- 0.03]) significantly predicted serum Metrnl (P < 0.05).

## Discussion

Obesity and adipose tissue dysfunction, as a part of MetS, are common in patients diagnosed with PCOS, adversely affecting their cardiometabolic function and systemic homeostasis through a disrupted release of adipokines. Intriguingly, insulin resistance has a bidirectional relationship with adipocyte dysfunction, and is an essential contributing factor in the trigger and exacerbation of PCOS pathogenesis. This study is the first to show that serum levels of Metrnl tend to be lower in patients diagnosed with PCOS and RPL, inversely correlating with markers of glucose homeostasis and inflammation.

The low serum levels of Metrnl in patients diagnosed with PCOS showed an independent association with the pathogenesis of the disease, partly through insulin resistance, as evidenced by the correlation between Metrnl levels and glucose homeostasis parameters. Similar correlation was observed in several studies [35–37]. For instance, Wang et al. found that Metrnl levels positively correlated with FBG, post-prandial BG, HbA1c, fasting insulin, and HOMA-IR. The authors regarded that Metrnl elevated the risk of T2DM, independent of insulin resistance [38]. Moreover, experimental studies have shown that Metrnl knockout models developed insulin resistance in response to high-fat diet (HFD); on the contrary, Metrnl overexpression in transgenic mice were able to antagonize it [28, 39]. A recent study by Lee et al. found that Metrnl improved glucose tolerance by increasing the phosphorylation of histone deacetylase 5 (HDAC5), thereby activating GLUT4 transcription, in an AMPKα2-dependent pathway, in HFD-induced obese or diabetic mice [34]. However, the methodology of the present study limits a causal relationship between lower levels of serum Metrnl and insulin resistance in PCOS setting. Nevertheless, it can be interpreted that these levels reflect the metabolic impairments observed in these patients.

Moreover, our results showed a significant decrement in serum Metrnl levels in overweight/obese individuals regardless of their disease status, when compared to controls [40, 41]. Lowest levels were observed in infertile obese patients diagnosed with PCOS. However, the association of serum Metrnl with obesity biomarkers is still paradoxical. For instance, El-Ashmawy et al. and other authors found no association between serum Metrnl and BMI [28, 42, 43]; while Al-Khairi at el. found a significant correlation between the two in obese subjects [37]. This paradox might be due to methodological differences in terms of obesity cutoffs, i.e. BMI exceeding 25 Kg/m^2^ or 30 Kg/m^2^. Nevertheless, stratifying individuals according to BMI alone has been regarded as “an imperfect predictor of body composition and disease risk, but still has some clinical value as a crude estimate”, posing the risk of misclassification [44]. For instance, Stefanaki et al. showed that lean PCOS females (BMI 18.5–24.9 kg/m^2^) exhibited an osteosarcopenic phenotype, characterized by low muscle mass and bone mineral density, but normal total and subcutaneous fat mass [45]. A recent study by Du et al. found that serum Metrnl levels negatively correlated with visceral adiposity (exceeding 100 cm^2^) in diabetic patients (OR = 0.846, 95% CI [0.745–0.961], P = 0.01) [46]. Therefore, there is a need to assess the functional body composition of PCOS subjects to have a better image on the association of fat mass and fat free mass with, in this case, serum Metrnl levels. In addition, physical activity could be a confounding factor, elevating the levels of Metrnl in obese and/or diabetic patients in these studies. Exercise training induces mitochondrial biogenesis and release of Metrnl through the upregulation of peroxisome proliferator-activated receptor-γ coactivator-1 (PGC-1), thereby, suppressing adipocyte dysfunction and insulin resistance [26, 47, 48].

An inverse correlation with serum TG was detected in the subgroup only. We could not detect any association between serum levels of Metrnl and other parameters of lipid profile, unlike previous studies [35, 46]. However, this might be explained by the effect of Metrnl treatment in attenuating HFD-induced hypertriglyceridemia in transgenic mice, but not hypercholesterolemia [23].

Interestingly, infertile PCOS patients tended to have lower levels of serum adiponectin, worse insulin resistance, and higher LH to FSH ratio, as well. On the other hand, PCOS-RPL patients had higher serum Metrnl, homocysteine, and TG. Nevertheless, they were able to conceive, unlike PCOS-Inf. It can be hypothesized that lower serum levels of Metrnl and adiponectin, and higher levels of homocysteine in patients diagnosed with PCOS, synergistically add to the ongoing inflammatory process and insulin resistance. Usually, adiponectin performs anti-inflammatory functions, insulin-sensitizing, and cardioprotective functions [49, 50] by inhibiting NF-κB pathway in macrophages and dendritic cells [51–53], and is involved in regulating fertility, ovarian functions, and cytotrophoblast invasion [54]. Hypoadiponectinemia has been associated with CVD [55], increased adiposity, impaired fasting glucose (IFG), insulin resistance, and DM [56], and PCOS [57, 58]. Surprisingly, it has pro-inflammatory function inducing the release of pro-inflammatory cytokines (such as TNF-α, IL-6, IL-1β) and prostaglandins (such as PG_2α_) in the placenta, by regulating NF-κB and PPAR-γ signaling pathway [59], in an attempt for an essential degree of inflammation necessary to promote syncytialization and the invasive capacity of cytotrophoblast. Adiponectin to leptin ratio is diminished in PCOS [60] and is elevated in those diagnosed with RPL only [61]. Our data showed significantly lower adiponectin levels in the PCOS group and subgroups, when compared to controls, coinciding with the aforementioned. Therefore, both elevated and depressed levels of this adipokine can negatively affect fertility rates in females diagnosed with PCOS with/out RPL, increasing the risk of insulin resistance and inflammation.

Furthermore, homocysteine is a potent inhibitor of adipogenesis, stimulating AMPK and suppressing PPAR-γ signaling pathways; and it intensifies the effect of hyperandrogenism on cardiovascular risk in females diagnosed with PCOS [61]. PCOS group and subgroups had higher homocysteine levels when compared to control, having a direct association with insulin resistance [62]. This might be related to the inhibitory effect of homocysteine on adiponectin biosynthesis by primary adipocytes and PPAR-γ signaling by increasing the methylation of PPAR-α/γ gene promoter [63], further exacerbating the insulin resistance and inflammation [64].

The PCOS-Inf and PCOS-RPL females also had significantly higher levels of hs-CRP, an acute phase reactant primarily produced by IL-6-induced hepatocytes [65]. Numerous studies demonstrated a correlation between higher serum hs-CRP levels with cardiovascular events in patients diagnosed with coronary artery disease (CAD), T2DM [66], PCOS [67, 68], and RPL [69]. However, its use as a “powerful predictor of cardiovascular risk” is still controversial [70]; while some studies have agreed to this notion [71], a 9-year prospective study concluded that hs-CRP did not pose any additional effect on conventional risk factors [72]. Nevertheless, in this study, hs-CRP levels were significantly higher in patients diagnosed with PCOS, also coinciding with previous literature. Furthermore, a strong inverse correlation was detected between serum Metrnl and hs-CRP levels in the PCOS group and subgroups. The same association was detected by Dadmanesh et al. [36], and Li et al. [73], in the context of T2DM and CAD. El-Ashmawy et al. also found a negative correlation between serum Metrnl and hs-CRP levels, IL-6, TNF-α, and endothelial dysfunction markers (E-selectin and intracellular adhesion molecule-1) in DM patients. The authors concluded that depressed levels of Metrnl might “be a stimulus of subclinical inflammation and insulin resistance”, increasing the risk for developing CVD and DM [42]. These further emphasize that Metrnl protein is involved in the inflammatory process. Metrnl biosynthesis is induced by an inflammatory stimulus (as by TNF-α levels), playing a regulatory role in the inflammatory response, upregulating the production of anti-inflammatory cytokines (such as IL-4) and abrogating the production of pro-inflammatory cytokines and chemokines [74]. Metrnl might normally balance the anti-inflammatory effect of adiponectin to control the inflammatory response and induce the action of immunoregulatory immune cells, just enough to create a harboring medium for the approaching fertilized egg. However, in the case of PCOS-Inf, serum levels of Metrnl were not enough to break the shield of inflammation created by hypoadiponectinemia and hyperhomocysteinemia. Therefore, Metrnl protein might have served a protective effect, synergistically with adipokines such as adiponectin, CTRP 12 & 13 [75], against the inflammation and insulin resistance present in PCOS-RPL group, through PPAR-γ signaling pathway, but was not enough to maintain viable pregnancy. Future studies might include assessing the expression of PPAR-γ and its association with Metrnl protein in the pathogenesis of PCOS and RPL to get a better picture of the underlying mechanisms.

Currently, this is the first study to inquire serum Metrnl protein in females diagnosed with PCOS and/or RPL. However, there are limitations. First, we could not exclude all potential bias and confounding factors such as exercise training. Second, in this study, we have used BMI for assessing body composition based on statistical criterion, and the use of body composition analysis (dual energy X ray absorptiometry (DEXA) and bioelectrical impedance analysis (BIA)) and functional body composition can give better insight on the possible relation between serum Metrnl levels and the different tissue masses [44]. Third, additional assessment of clinical parameters could have been beneficial at drawing more exact conclusions, such as Ferriman score, and ovarian volume. Fourth, insulin resistance was calculated using HOMA-IR, instead of the gold standard, the euglycemic/hyperglycemic clamp. Nevertheless, HOMA-IR remains a universally accepted marker of insulin resistance. Moreover, an additional group of females affected by RPL but not PCOS could have shed more light on the association of serum Metrnl with the pathogenesis of recurrent miscarriage.

The results of the present study revealed an independent association between Metrnl protein and PCOS, with a negative correlation with insulin resistance and inflammation. Therefore, Metrnl might play a role in the pathogenesis of PCOS and recurrent miscarriage. Nevertheless, the study design limits further conclusion about the consequential relation between Metrnl and the diseases.

## Supporting information

S1 Table Clinical features of the normal (< 25 Kg/m^2^) vs overweight/obese (≥ 25 Kg/m^2^) patients. (DOCX)

S1 File Free testosterone ELISA kit. (DOCX)
