# Supplementary material for: Circulating levels of Meteorin-like protein in polycystic ovary syndrome: A case-control study
Source: PLoS One. 2020 Apr 24;15(4):e0231943. doi: 10.1371/journal.pone.0231943 (PMC7182262; doi:10.1371/journal.pone.0231943)
Supplement: S1 Table — (DOCX) [file pone.0231943.s001.docx]

**Supporting information**

| **S1 Table. Clinical features of the Normal (< 25 Kg/m^2^) vs Overweight/Obese (≥ 25 Kg/m^2^) Patients** | | | | | | | | | |
| --- | --- | --- | --- | --- | --- | --- | --- | --- | --- |
| **Variables** | **Control**  **(n = 60)** | | **PCOS**  **(n = 120)** | | **PCOS-Inf**  **(n = 60)** | | **PCOS-RPL**  **(n = 60)** | | **p-value** |
|  | **Normal** | **Overweight** | **Normal** | **Overweight** | **Normal** | **Overweight** | **Normal** | **Overweight** |  |
| **BMI (Kg/m^2^)** | 22.41 ± 1.75 | 27.67 ± 2.11^**^ | 22.89 ± 1.64 | 28.40 ± 2.22^**^ | 22.61 ± 1.95 | 28.35 ± 2.18^**^ | 23.16 ± 1.24 | 28.46 ± 2.29^**^ | < 0.001 |
| **HDL-C (mg/dL)** | 46.32 ± 5.81 | 45.93 ± 7.67 | 46.87 ± 12.15 | 43.56 ± 7.61 | 45.15 ± 9.42 | 44.76 ± 8.22 | 48.60 ± 14.36 | 42.36 ± 6.85^*^ | < 0.05 |
| **FSH (IU/L)** | 6.49 ± 2.64 | 6.38 ± 2.25 | 7.73 ± 4.21 | 6.43 ± 2.27^*^ | 8.55 ± 5.28 | 5.81 ± 2.13^*^ | 6.91 ± 2.62 | 7.05 ± 2.26 | < 0.05 |
| **Log Fasting Insulin (μU/mL)** | 0.94 ± 0.51 | 1.16 ± 0.55 | 1.27 ± 0.59 | 1.49 ± 0.59^*^ | 1.20 ± 0.47 | 1.51 ± 0.48^*^ | 1.34 ± 0.69 | 1.46 ± 0.69 | < 0.05 |
| **Log Adiponectin** | 1.79 ± 0.44 | 1.49 ± 0.51^*^ | 1.06 ± 0.43 | 0.83 ± 0.44^*^ | 1.04 ± 0.41 | 0.74 ± 0.39^*^ | 1.07 ± 0.46 | 0.93 ± 0.46 | < 0.05 |

Parametric variables are given as mean ± standard deviation

Non-parametric variables are given as median and interquartile range [25—75%]

Post Hoc Analysis are given as mean and median of each group on the far-right column

* P < 0.05 is of statistical significance

** P < 0.001 is of statistical significance

PCOS: Polycystic ovary syndrome; PCOS-Inf: Infertile PCOS; RPL: Recurrent pregnancy loss; BMI: Body mass index; HDL-C: high density lipoprotein cholesterol; FSH: follicle-stimulating hormone

**Free Testosterone ELISA Kit**

In the present study, we relied on a good quality ELISA kit (Monobind, U.S.A., minimal detection concentration 0.04 pg/mL, intra-assay CV 8.9%, inter-assay CV 12.4%) to measure testosterone levels in the female subject. The kit was calibrated against the Siemens Free Testosterone RIA kit (51 serum samples were analyzed, linear regression curve was calculated as Y = 1.81 * X – 1.71, r-squared = 0.94).
