# Supplementary material for: Circulating levels of Meteorin-like protein in polycystic ovary syndrome: A case-control study
Source: PLoS One. 2020 Apr 24;15(4):e0231943. doi: 10.1371/journal.pone.0231943 (PMC7182262; doi:10.1371/journal.pone.0231943)
Supplement: S1 File — (DOCX) [file pone.0231943.s002.docx]

**Free Testosterone ELISA Kit**

In the present study, we relied on a good quality ELISA kit (Monobind, U.S.A., minimal detection concentration 0.04 pg/mL, intra-assay CV 8.9%, inter-assay CV 12.4%) to measure testosterone levels in the female subject. The kit was calibrated against the Siemens Free Testosterone RIA kit (51 serum samples were analyzed, linear regression curve was calculated as Y = 1.81 * X – 1.71, r-squared = 0.94).
